# Inhibitors of bacterial protease enzymes for periodontal therapy

**DOI:** 10.1002/cre2.4

**Published:** 2015-10-27

**Authors:** Kalid N. Hosn, Mary Margaret Jefferson, Carlton Leding, Solomon Shokouh‐Amiri, Edwin L. Thomas

**Affiliations:** ^1^ Periodontology Department University of Tennessee Health Science Center Memphis Tennessee USA; ^2^ Bioscience Research Department, College of Dentistry University of Tennessee Health Science Center Memphis Tennessee USA; ^3^ Microbiology, Immunology & Biochemistry Department University of Tennessee Health Science Center Memphis Tennessee USA

**Keywords:** Microbial viability, periodontal diseases, *Porphyromonas gingivalis*, protease inhibitors

## Abstract

Locally applied therapeutic agents have become established in the treatment of periodontal disease. Inhibition of human metalloproteases by metal‐chelating antibiotics contributes to the utility of local therapy. Adding inhibitors of bacterial proteases might extend and improve local therapy. The periodontal pathogen *Porphyromonas gingivalis* (Pg) produces two extracellular cysteine proteases (gingipains Rgp and Kgp) that are virulence factors and contribute to destruction of oral tissues. Our aims were to compare efficacy of protease inhibitors against gingipains and evaluate bactericidal activity of the inhibitors. Protease activity was measured in fluorescent assays with specific Rgp and Kgp substrates. Bacterial viability was measured with *Bac*Light™ (Invitrogen, Inc., Carlsbad, CA) reagents. Pairs of inhibitors of Rgp and Kgp, respectively, were leupeptin and cathepsin B inhibitor II, KYT‐1 and KYT‐36, and PPACK and Z‐FK‐ck. The cysteine‐protease inhibitor E64 was also tested. Rgp activity was higher than Kgp activity, and activity was higher in Pg 33277 and 49417 cell suspensions than in media. Concentrations required for 50% inhibition of Rgp in cell suspensions were 2 × 10^−9^, 2 × 10^−9^, 2 × 10^−8^, and 5 × 10^−5^ M for KYT‐1, PPACK, leupeptin, and E64, respectively. Concentrations required for 50% Kgp inhibition were 5 × 10^−10^, 1 × 10^−9^, and 5 × 10^−8^ M for Z‐FK‐ck, KYT‐36, and cathepsin B inhibitor II. E64 did not inhibit Kgp. Inhibition of Rgp could be accounted for by competition for binding between the arginine residue of the substrate and the guanidinobutane portion of E64. PPACK was the least selective, with a 10‐fold difference in concentrations that inhibited Rgp and Kgp. KYT‐1 and Z‐FK‐ck inhibited both Rgp and Kgp, but inhibitory concentrations differed by 10,000‐fold. At up to 1 × 10^−4^ M, only Z‐FK‐ck was bactericidal. KYT‐1 and KYT‐36 were remarkably effective even when used in cell suspensions in which bacterial proteins could bind inhibitors or compete for binding to gingipains. These inhibitors might prove useful as an addition to locally applied therapeutic agents.

## Introduction

New advanced surgical techniques and materials and new approaches to delivering local antimicrobial agents as a monotherapy or as adjuncts to non‐surgical therapy have improved the outcome and prognosis in the treatment of periodontal disease or prevention of recurrence (Krayer et al., 2010). These agents include chlorhexidine chip (Jeffcoat et al., 1998), tetracycline‐containing fibers (Goodson et al., 1979; Tonetti, 1998), and subgingival doxycycline (Larsen, 1991), minocycline (Williams et al., 2001), and metronidazole (Stelzel & Flores‐De‐Jacoby, 1996).

The utility of the tetracyclines including doxycycline and minocycline is due not only to antibacterial activity but also to the ability of these metal‐chelating agents to inhibit tissue matrix metalloproteases (MMPs) that contribute to hydrolysis of connective tissue components (Gu et al., 2011). Doxycycline can also be taken orally as a systemic adjunct to periodontal therapy. It is used at a low level that inhibits MMPs but does not have antimicrobial activity and thus does not select for antibiotic‐resistant bacteria (Caton & Ryan, 2011).

### Periodontitis and periodontal pathogens

Periodontitis is characterized by destruction of tooth‐supporting soft and hard tissues of the periodontium, including cementum, alveolar bone, and periodontal ligament, owing to a complex interaction between the subgingival biofilm and the host immune system in response to the invading periodontal pathogenic bacteria.

Subgingival bacterial complexes as described by Socransky et al. in 1998 (Socransky et al., 1998) include the red complex of *Bacteroides forsythus* (since renamed *Tannerella forsythia*), *Porphyromonas gingivalis* (Pg), and *Treponema denticola*, which produce a wide variety of metabolic by‐products and pro‐inflammatory mediators that cause inflammatory tissue injury. Plaque bacteria also produce proteases, which can break down structural proteins of the periodontium such as collagen, elastin, and fibronectin (Bostanci & Belibasakis, 2012). Bacteria produce these proteases to digest proteins and thereby obtain nutrients including peptides, amino acids, and iron. Bacterial proteases also disrupt host responses, compromise tissue integrity, and facilitate microbial invasion of the tissues. Andrian et al. (2006) reported that periodontal pathogens such as Pg have developed strategies to perturb the structural and functional integrity of the gingival epithelium. Pg also subverts host responses to bacterial challenges by inactivating immune cells and activating host processes leading to tissue destruction (Potempa & Pike, 2009).

Pg produces two classes of proteases known as gingipains that have the amino acid residue cysteine at the active site and which hydrolyze proteins at the bond between arginine (R) or lysine (K) and the next residue in the protein sequence (Pike et al., 1994). The arginine‐specific gingipains RgpA and RgpB and lysine‐specific gingipain Kgp have been implicated in periodontal pathogenesis. Gingipains can modulate the immune system and disrupt immune‐inflammatory responses, potentially leading to increased tissue breakdown (Potempa & Pike, 2009; Baba et al., 2002). Gingipains can reduce the concentrations of cytokines in cell culture systems, and they digest and inactivate TNF‐α (Calkins et al., 1998). The gingipains can also stimulate cytokine secretion via activation of protease‐activated receptors (Giacaman et al., 2009). Another possible role for gingipains in pathogenesis is hydrolysis and inactivation of antimicrobial proteins and peptides of leukocytes and epithelial cells (Potempa & Pike, 2009; Maisetta et al., 2011; McCrudden et al., 2013).

Evidence for gingipain activity in oral tissues is limited but supported by studies that detected products of gingipain‐mediated proteolysis of oral tissue components in gingival crevicular fluid of periodontitis patients (Ruggiero et al., 2013; Tancharoen et al., 2015).

### Aims

One aim of this study was to compare the efficacy of protease inhibitors against Rgp and Kgp as a step toward evaluating their possible use as components of locally applied therapeutic agents. Experiments were carried out with culture media and resuspended Pg cells rather than with purified gingipains, to take into account the ability of bacterial components to bind or inactivate the inhibitors or to compete with the inhibitors for binding to the gingipain active sites.

The specificity of inhibitors for Rgp and Kgp was also examined. Although it is likely that inhibition of both Rgp and Kgp would be desired in clinical applications, it would be helpful to determine whether inhibition of Rgp or Kgp or both is needed for a clinical effect and to determine the effective concentration of each inhibitor.

Finally, bactericidal activity of the inhibitors was examined. Gingipain inhibitors without bactericidal activity would be preferred for clinical applications to avoid selecting for inhibitor‐resistant mutants.

### Gingipain inhibitors

Three pairs of inhibitors of Rgp and Kgp were compared: leupeptin and cathepsin B inhibitor II, KYT‐1 and KYT‐36, and PPACK and Z‐FK‐ck. The cysteine protease inhibitor E64 was also tested.

Leupeptin is a bacterial product that inhibits many serine, threonine, and cysteine proteases (Bogyo & Wang, 2002). It is an acetylated tripeptide with a C‐terminal aldehyde rather than a carboxyl group, represented as Ac‐Leu‐Leu‐Arg‐aldehyde or Ac‐LLR‐CHO. It is a competitive inhibitor of many proteases including the cysteine‐protease cathepsin B and the serine‐protease trypsin. Leupeptin was reported to inhibit Rgp but not Kgp (Pike et al., 1994; Smalley et al., 2007). Cathepsin B inhibitor II is a similar synthetic tripeptide represented as Ac‐Leu‐Val‐Lys‐aldehyde or Ac‐LVK‐CHO (McConnell et al., 1993). It is almost 100 times more effective than leupeptin as an inhibitor of cathepsin B (McConnell et al., 1993) and was reported to inhibit Kgp but not Rgp (Grenier et al., 2001).

KYT‐1 and KYT‐36 were developed specifically as competitive inhibitors of purified Rgp and Kgp, respectively (Kadowaki et al., 2004). KYT‐1 is a chemically modified peptide with two lysine residues and one arginine residue, represented as carbobenzoxy‐Lys‐Arg‐CO‐Lys‐N(CH_3_)_2_. KYT‐1 was designed to take advantage of the specificity of Rgp, which prefers to cleave between arginine and lysine residues, especially when arginine is preceded by lysine. The additional carbonyl group between arginine and lysine prevents hydrolysis and inactivation of the inhibitor. KYT‐36 has one lysine and no arginine residues and is represented as carbobenzoxy‐Glu(NHN(CH_3_)Ph)‐Lys‐CO‐NHCH_2_Ph. KYT‐36 takes advantage of the specificity of Kgp, which prefers to cleave between lysine and histidine residues, especially when lysine is preceded by glutamic acid. In KYT‐36, the side chain of glutamic acid is modified, another group is substituted for histidine, and the additional carbonyl group after lysine prevents hydrolysis.

A highly effective irreversible inhibitor of Rgp is PPACK, represented as D‐Phe‐Pro‐Arg‐chloromethylketone or fPR‐CMK (Potempa et al., 1997). A similar inhibitor of Kgp is Z‐FK‐ck, represented as Z‐Phe‐Lys‐2,4,6‐trimethylbenzoyloxy‐methylketone (Potempa et al., 1997; Wagner et al., 1994). The methylketone groups chemically modify the gingipain active sites. In one study, PPACK was used to inactivate both Rgp and Kgp (Potempa et al., 1997).

E64 (*trans*‐epoxysuccinyl‐l‐leucylamido‐(4‐guanidino)butane) is a fungal product that inactivates many cysteine proteases (Barrett et al., 1982), although it also inhibits the serine‐protease trypsin (Sreedharan et al., 1996). E64 consists of a leucine residue with the amino group amide‐linked to epoxysuccinic acid and the carboxyl group amide‐linked to guanidinobutylamine, an analogue of arginine that lacks the carboxyl group. The epoxysuccinyl group of E64 reacts with thiol groups at the active site of cysteine proteases but does not react with free cysteine or cysteine residues of non‐proteolytic enzymes (Barrett et al., 1982). Several studies reported that E64 inhibited Rgp (Rangarajan et al., 1997; Kadowaki et al., 1994; Chen et al., 1992). In another study, E64 was used to partially inhibit Pg proteolytic activity that was not due to gingipains (Potempa et al., 1997).

## Materials and Methods

### Protease inhibitors

Leupeptin and cathepsin B inhibitor II were purchased from Sigma‐Aldrich (St. Louis, MO) and Calbiochem (Billerica, MA), respectively. PPACK and Z‐FK‐ck were purchased from Bachem Americas, Inc. (Torrance, CA). KYT‐1 and KYT‐36 were purchased from PeptaNova, GmbH (Sandhausen, Germany). Unless otherwise indicated, other reagents including protease inhibitors E64, *N*‐α‐tosyl‐l‐lysine chloromethyl ketone HCl (TLCK), and phenylmethanesulfonyl fluoride (PMSF) were purchased from Sigma‐Aldrich.

Stock solutions of leupeptin (10 mM), cathepsin B inhibitor II (3 mM), and E64 (10 mM) were prepared in water, and stock 10 mM solutions of PPACK, Z‐FK‐ck, KYT‐1, and KYT‐36 were prepared in dimethyl sulfoxide (DMSO). All were stored in small aliquots at −80°C. PMSF (0.1 mM) was prepared fresh in dry methanol. TLCK (1.5 mM) was prepared on the day of use in 1 mM HCl.

### Gingipain substrates

Benzoyl‐R‐AMC and Boc‐VLK‐AMC were purchased from Sigma‐Aldrich and Bachem Americas, respectively. Stock 10 mM solutions of each were prepared in DMSO and stored at −80°C in 50 μL aliquots. For use, solutions were thawed and diluted to 2 mM with Dulbecco's phosphate‐buffered saline (DPBS) without calcium or magnesium (Life Technologies, Carlsbad, CA).

### Bacterial strains and culture conditions

Pg strains 33277 and 49417 were purchased from the American Type Culture Collection (Manassas, VA). These strains differed in their ability to grow in a defined medium with a single protein as a source of nutrients, implying a difference in their ability to digest the protein or to utilize the resulting peptides and amino acids (Leduc et al., 1996). The bacteria were grown overnight at 37°C under anaerobic conditions in trypticase soy broth (TSB) supplemented with yeast extract (1%), both from Becton, Dickenson & Company (Sparks, MD), and with hemin (5 µg/mL) and menadione (1 µg/mL). Cultures were serially diluted in sterile DPBS and plated on BBL™ TSA II™ 5% sheep blood agar plates (Becton, Dickenson & Company) to confirm their identity as black‐pigmented colony formers on blood agar and to determine the number of colony‐forming units (CFU) per milliliter.

To obtain culture media for measuring secreted gingipain activity, we removed bacteria by centrifugation followed by filtration through 0.2‐µm filters, and we supplemented the medium with 0.2 mM PMSF to block any serine‐protease activity. To measure cell‐associated activity, we resuspended bacteria to an optical density of 4.0 at 600 nm (4 × 10^9^ CFU/mL) in DPBS, and PMSF was added. Portions of the media and bacterial suspensions were frozen in liquid nitrogen and stored at −80°C.

*Escherichia coli* strain 43827 was purchased from the American Type Culture Collection and grown in TSB overnight with shaking in air at 37°C. Suspensions were serially diluted in saline and plated on TSB agar to determine CFU per milliliter.

### Gingipain activity and effects of inhibitors

The gingipain assay was adapted from procedures of Chen et al. (2001) and Suwannakul et al. (2010). To measure activity of Rgp or Kgp, respectively, we incubated various volumes of media or bacterial suspensions 10 min at 37°C in DPBS with 1 mM l‐cysteine to ensure that the gingipain active‐site cysteine was in the reduced, active form, and then we added the substrate benzoyl‐R‐AMC or Boc‐VLK‐AMC to give 0.2 mM substrate in a final volume of 0.1 mL. The incubation was continued 15 min at 37°C in black multiwell plates. Incubations were stopped by adding 50 μL of 1.5 mM TLCK, an irreversible inhibitor of serine and cysteine proteases. Fluorescence was measured with a fluorescence plate reader (SpectraMax Gemini EM, Molecular Devices, Sunnyvale, CA) with excitation at 365 nm and emission at 460 nm to measure hydrolysis of the substrate and release of the fluorescent product.

To measure effects of inhibitors on Rgp or Kgp activity, we diluted conditioned media fivefold or bacterial suspensions fiftyfold with DPBS, and we selected a diluted sample volume of 20 μL or less that gave a strong fluorescent signal, and we incubated the sample 15 min in DPBS with 0.2 mM substrate and various levels of protease inhibitors. Control experiments indicated that assays were linear with respect to time and sample volume under these conditions.

### Bactericidal activity of protease inhibitors

Pg bacteria were collected by centrifugation and diluted to 2 × 10^8^ CFU/mL in isotonic saline at 25°C. Pg (10^8^ CFU/mL) were then incubated with various concentrations of protease inhibitors at 37°C with gentle mixing in 0.1 mL incubation mixtures in black multiwell plates inside AnaeroGen™ Compact pouches (Oxoid Corporation, Nepean, ON, Canada) to lower the oxygen (O_2_) concentration. After 1 h, the number of live bacteria per milliliter was measured with a LIVE/DEAD *Bac*Light™ Bacterial Viability kit (Invitrogen, Inc., Carlsbad, CA). The assay determines the number of live bacteria based on entry of the fluorescent dyes SYTO 9 and propidium iodide into the bacteria. SYTO 9 enters both live and dead bacteria and binds to DNA, giving a green fluorescence. Propidium iodide enters only dead bacteria and binds to DNA, giving a red fluorescence. A 0.1 mL portion of 2× *Bac*Light dye mixture was added to the bacteria in the wells, and the plate was incubated 15 min at 37°C in an AnaeroGen™ pouch in the dark. Immediately following removal of the plate from the pouch, fluorescence was measured in a fluorescence plate reader with excitation at 485 nm. Green fluorescence of SYTO 9 was measured at 538 nm, and red fluorescence of propidium iodide was measured at 612 nm. Standard curves were prepared with various ratios of live and dead bacteria, and the number of live bacteria was determined from the ratio of green to red fluorescence. Control experiments were performed in which Pg viability was measured by diluting, plating on blood agar plates, and counting colonies after 1 to 2 weeks at 37°C under anaerobic conditions to confirm that the plating and fluorescent assays provided similar results. Toxicity of the protease inhibitors to *E*. *coli* was also measured. Assay conditions were the same as for Pg except that incubations were in air.

### Data analysis

All experiments were performed in triplicate with duplicate incubation mixtures. Statistical significance was evaluated by analysis of variance followed by Scheffé's *f* test to calculate *P* values. Values <0.05 were considered significant.

## Results

### Gingipain activity in cells and media

Gingipain activity was measured in cell suspensions and culture media for both Pg strains (Table 1). Activity in cell suspensions was much higher than in media for both Rgp and Kgp in both Pg strains. Although Rgp and Kgp were first isolated from culture media (Pike et al., 1994; Chen et al., 1992), the enzymes undergo extensive post‐translational modification that results in attachment of most of the Rgp and Kgp to the Pg outer membrane (Zhou et al., 2013).

**Table 1:** Gingipain activity in cells and media.

	(10^3^ RFU/min/mL)		
	Cells	Media	Total
Rgp activity			
Pg 33277	1820	65	1885
Pg 49417	643	80	722
Kgp activity			
Pg 33277	825	6	831
Pg 49417	293	26	319

Activities in relative fluorescence units (RFU)/min/mL were calculated from the slope of plots of RFU versus four volumes of cell suspension or culture media. Values were corrected for dilution of the cells or media relative to the original culture and were divided by time of the 15‐min incubation. Data from all experiments were plotted in single graphs, and the slopes were determined by linear regression. The linear correlation coefficient, *r*, was ≥0.9 for all slopes except for Kgp activity of Pg 33277 cells, which was 0.83.

Table 1 also shows that total Rgp activity in cells and media was about 2.3 times higher than Kgp activity in both strains. A ratio of Rgp to Kgp of between 2 and 3 was determined by measuring the number of active sites (Potempa et al., 1997). Our results are consistent with those findings, assuming the Rgp and Kgp enzymes have similar turnover numbers.

**Figure 1:**
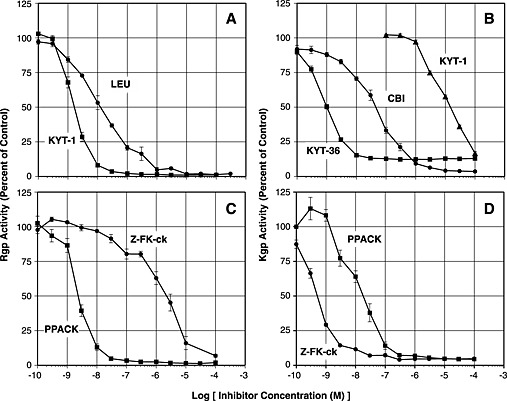
Inhibition of gingipain activity. Rgp activity (plots A and C) and Kgp activity (plots B and D) of Pg 33277 cell suspensions were plotted as percent of the control (without inhibitor) versus the log of the concentrations of competitive inhibitors [A: KYT‐1 (■), leupeptin (LEU, ●); B: KYT‐36 (■); cathepsin B inhibitor II (CBI, ●); KYT‐1 (▲)] or irreversible inhibitors [plots C and D: PPACK (■); Z‐FK‐ck (●)].

**Figure 2:**
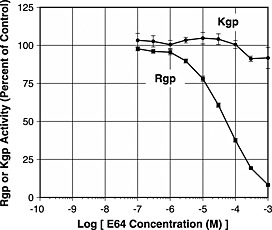
Gingipain activity in the presence of E64. Rgp activity (■) and Kgp activity (●) of Pg 33277 cell suspensions were plotted as percent of control (without E64) versus the log of E64 concentration.

Because more activity was found in cell suspensions than in culture media, it was more convenient to compare inhibition of activity in suspensions, and Figures 1 and 2 show results obtained with suspensions. Experiments were also carried out with culture media, and comparable results were obtained.

Similarly, it was more convenient to use the higher activity 33277 strain, and results with this strain are shown in Figures 1 and 2. However, all experiments were carried out with both strains, and no significant differences were observed between the 33277 and 49417 strains.

### Reversible competitive inhibitors

Figure 1 (top) shows inhibition of Rgp and Kgp activities in cell suspensions by various concentrations of two pairs of competitive inhibitors. KYT‐1 and KYT‐36 were very effective inhibitors, causing 50% inhibition of Rgp and Kgp activity at 2 × 10^−9^ and 1 × 10^−9^ M, respectively. These inhibitors were more effective than leupeptin and cathepsin B inhibitor II, which caused 50% inhibition of Rgp and Kgp activities at 2 × 10^−8^ and 5 × 10^−8^ M, respectively.

Results in Figure 1 (top) show that KYT‐1 inhibited both Rgp and Kgp. KYT‐1 caused 50% inhibition of Kgp at 2 × 10^−5^ M, which is 10,000‐fold higher than the concentration that caused 50% inhibition of Rgp. In contrast, KYT‐36 was specific for Kgp and did not inhibit Rgp at concentrations up to at least 1 × 10^−4^ M (results not shown). Similarly, cathepsin B inhibitor II and leupeptin at concentrations up to 1 × 10^−4^ M had no effect on Rgp and Kgp, respectively (results not shown).

### Irreversible inhibitors

Figure 1 (bottom) shows inhibition of Rgp and Kgp activities in cell suspensions by various concentrations of the pair of irreversible inhibitors. PPACK was the least selective of the inhibitors tested. PPACK caused 50% inhibition of Rgp and Kgp at 2 × 10^−9^ and 2 × 10^−8^ M, respectively, a 10‐fold difference in concentration. Z‐FK‐ck inhibited both enzymes but was much more effective against Kgp. Z‐FK‐ck caused 50% inhibition of Rgp and Kgp at 2 × 10^−6^ and 5 × 10^−10^ M, respectively, nearly a 10,000‐fold difference in concentration.

### Inhibition by E64

Figure 2 shows that E64, an irreversible inhibitor of many cysteine proteases, was only a weak inhibitor of Rgp activity, causing 50% inhibition at 5 × 10^−5^ M and 90% inhibition at 1 × 10^−3^ M. E64 had no effect on Kgp activity at any concentration tested.

### Bactericidal activity

**Figure 3:**
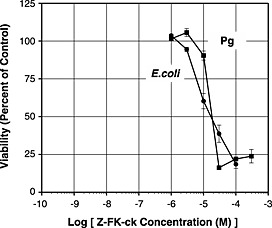
Bactericidal activity of Z‐FK‐ck. Viability of Pg 33277 (■) or *E*. *coli* (●) was plotted as percent of control (without Z‐FK‐ck) versus the log of Z‐FK‐ck concentration.

All of the inhibitors were incubated with both of the Pg strains and with *E*. *coli*. Only Z‐FK‐ck had significant bactericidal activity at concentrations up to 1 × 10^−4^ M. Figure 3 shows that a similar bactericidal activity was obtained with Pg or *E*. *coli*. Z‐FK‐ck caused a 50% loss of viability at 3 × 10^−5^ M, a concentration much higher than required to inhibit Rgp or Kgp. The similar results obtained with Pg and *E*. *coli* indicate that bactericidal activity was unrelated to inhibition of gingipains.

## Discussion

Adding gingipain inhibitors to locally applied therapeutic agents might extend and improve this form of therapy for periodontal disease. Of the inhibitors tested in this study, the competitive inhibitors KYT‐1 and KYT‐36 appeared to have the best combination of properties for clinical studies.

KYT‐1 and KYT‐36 were effective inhibitors of Rgp and Kgp activities, respectively, and were effective at lower concentrations than the competitive inhibitors leupeptin and cathepsin B inhibitor II. KYT‐1 and KYT‐36 were effective even in cell suspensions in which bacterial components might bind the inhibitors and lower their concentrations.

KYT‐1 and KYT‐36 may also be more specific inhibitors of gingipains, in that their structures were designed to mimic sites in peptides that are especially susceptible to hydrolysis by gingipains (Kadowaki et al., 2004). Specific inhibition of gingipains would be an advantage because there are many proteases in human tissues that are required for normal function of oral tissue cells and phagocytic leukocytes.

KYT‐1 and KYT‐36 were reported to be non‐toxic to cultured human cells (Kadowaki et al., 2004; Kataoka et al., 2014). In our study, KYT‐1 and KYT‐36 were also not bactericidal. Although bactericidal agents might seem to be preferred for periodontal therapy, protease inhibitors with bactericidal activity might select for inhibitor‐resistant mutant bacteria, especially when the agents are used over long periods of time. Mutants might produce gingipains with altered structures that do not bind the inhibitors or might produce substances that bind or inactivate inhibitors.

The possibility of selecting for resistant mutants has been a continuing concern in the use of local and systemic antimicrobial agents in periodontal therapy. The transient appearance of resistant bacteria was detected in a clinical study (Rodrigues et al., 2004). New approaches to avoid selecting for resistant bacteria were recently reviewed (Mombelli, 2012).

It should be noted that any protease inhibitor that inhibits bacterial growth would exert a selective pressure that might lead to resistance. If gingipain activity is required for the bacteria to obtain nutrients such as amino acids and iron, then growth will be slowed when gingipains are inhibited. Resistant mutants would have an advantage and would out‐compete susceptible bacteria.

At this time, KYT‐1 and KYT‐36 do not appear to have been tested in a periodontitis model. An analogue (KYT‐41) with structures similar to those in both KYT‐1 and KYT‐36 has been tested in animal models with positive results (Kataoka et al., 2014). On the other hand, a combination inhibitor might not be the best choice. Unless the bridge between the two active portions of the inhibitor is very long, one molecule of inhibitor could not bind simultaneously to the active sites of two enzyme molecules. Therefore, the inhibitor concentration needed to inhibit both Rgp and Kgp would be higher than the concentrations of two separate inhibitors. Moreover, for clinical studies, it would be desirable to test Rgp and Kgp inhibitors separately and together to determine whether both are needed, and if so, the optimum ratio of inhibitors.

The subject of inhibitors of gingipains for possible clinical use was recently reviewed (Olsen & Potempa, 2014), and KYT‐1 and KYT‐36 were cited as the best inhibitors currently available.

In our study, the irreversible inhibitor PPACK was the least selective. Although PPACK would be useful as a single inhibitor of Rgp and Kgp, it would not be possible to determine whether any clinical benefit was a result of inhibiting Rgp or Kgp. The irreversible inhibitor Z‐FK‐ck was especially effective against Kgp at low concentrations but has the disadvantage of being bactericidal, which could select for resistance. Moreover, if Z‐FK‐ck is toxic to bacteria, it may also be toxic to human cells.

Inhibitors like PPACK and Z‐FK‐ck that chemically modify proteins are not favored as pharmacologic agents in that chemical modification may create new epitopes that lead to production of antibodies to the protein. This would not be a problem if only bacterial proteins were modified, but these inhibitors might also react with host proteins.

E64 would not be suitable as a locally applied therapeutic agent for periodontitis. E64 did not inhibit Kgp and was only a weak inhibitor of Rgp. Inhibition of Rgp could be accounted for by competition between the guanidinobutane portion of E64 and the arginine residue of the substrate benzoyl‐R‐AMC. The results indicate that gingipains are not members of the class of cysteine proteases that are inactivated by E64.

On the other hand, E64 analogues with arginine and lysine residues in place of leucine might be effective gingipain inhibitors. Many E64 analogues have been synthesized and tested as inhibitors of cysteine proteases (Greenbaum et al., 2000), but inhibition of gingipains has not been tested at this time.

Further studies are needed to evaluate any toxicity of KYT‐1 and KYT‐36 or other inhibitors to cultured human oral tissue cells and to evaluate any clinical benefit of the inhibitors in animal models of periodontitis. Because KYT‐1 and KYT‐36 are water soluble, the inhibitors must be formulated in a similar manner to the antibiotics that are in current use to allow local placement, slow release, and persistent effects.

## Conflict of Interest

The authors have nothing to disclose.
